# Overcoming Detection Challenges of 2,4‑D Herbicides via SERS through a Simple Modification of Citrate-Reduced Silver Nanoparticles

**DOI:** 10.1021/acsomega.5c03570

**Published:** 2025-08-05

**Authors:** Guilherme Dognani, Francisca Belen Fuenzalida, Carlos José Leopoldo Constantino, Santiago Sanchez-Cortes

**Affiliations:** † School of Science and Technology, 28108 São Paulo State University (UNESP), Presidente Prudente, 19060-900 São Paulo, Brazil; ‡ Faculty of Science, Pavol Jozef Safarik University, 040 01 Kosice, Slovakia; § Instituto de Estructura de la Materia (IEM-CSIC), E-28006 Madrid, Spain

## Abstract

The difficulty in detecting certain pesticides at low concentrations in aqueous media made it necessary to search for new strategies to facilitate the detection of these contaminants. In this context, surface-enhanced Raman scattering (SERS) is a promising technique capable of carrying out the detection of hard-to-detect molecules. A pesticide called 2,4-dichlorophenoxyacetic acid (2,4-D) is within the group of these molecules that is difficult to detect. Currently, 2,4-D is recognized as one of the main herbicides used around the world, which has attracted the attention of researchers. This study investigates the synthesis of silver nanoparticles using a modified method based on citrate reduction. Three different colloids, AgCit_1.0_, AgCit_0.50_, and AgCit_0.25_, were synthesized with varying concentrations of citrate reductant. UV–vis extinction spectroscopy confirmed the formation of silver nanoparticles, exhibiting plasmon peaks at 405, 414, and 417 nm for AgCit_1.0_, AgCit_0.50_, and AgCit_0.25_, respectively. The SERS effect demonstrated the impact of citrate concentration on signal intensity and revealed characteristic peaks associated with citrate and the pesticide 2,4-D. The results demonstrated that there is a cutoff range where lower citrate concentrations (AgCit_0.50_ and AgCit_0.25_) presented higher limits of detection (LOD) values compared with the traditional silver-citrate nanoparticle (AgCit_1.0_). Therefore, the colloids AgCit_0.50_ and AgCit_0.25_ present a LOD by the signal/noise method of 1.85 × 10^–7^ and 1.20 × 10^–7^ mol/L, respectively, while AgCit_1.0_ showed a LOD of 3.10 × 10^–6^ mol/L. Linear regression confirms the LOD cutoff values. Thus, it is shown that the variation in citrate has an effect on the detection of the present pesticide.

## Graphical abstract

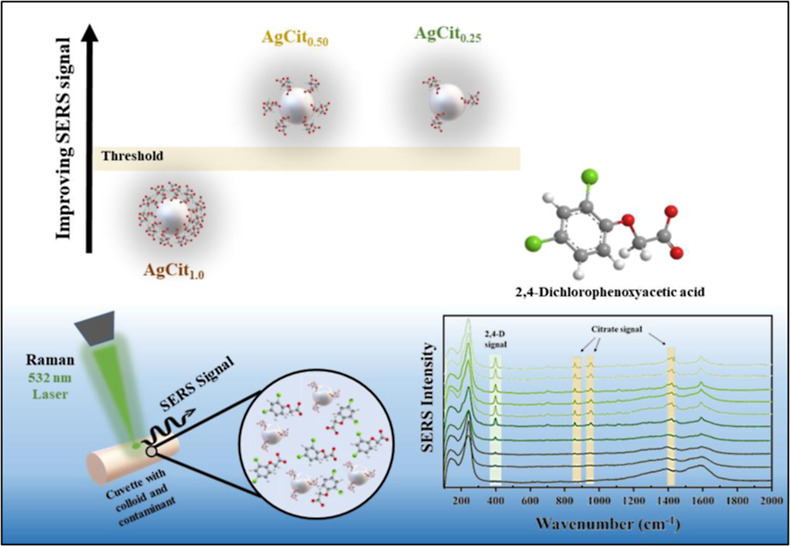

## 1 Introduction

The extensive use of pesticides in modern agriculture, considered by farmers as essential for pest control and increased crop yields, has raised huge concerns about environmental pollution.^1,2^ The widespread application of these chemical compounds may represent a significant risk to ecosystems, water bodies, and nontarget organisms.^3^ Pesticides can contaminate soil and water through runoff, potentially reaching ground areas and affecting aquatic life.^4,5^ The persistence of certain pesticides leads to long-term environmental consequences, as these contaminants may accumulate in the environment and pose a threat to biodiversity.^6,7^ The accumulation of pesticide residues in plant-based food, fish, and water sources extends the reach of these pollutants, potentially impacting human health.^8–10^ As the global demand for food production continues to rise, finding a balance between effective agricultural management and mitigating the environmental impact of pesticides remains a critical challenge for sustainable agriculture.^11^

The detection of pesticides at low concentrations is still important for environmental protection, as even small amounts can cause environmental damage. Accurate and sensitive pesticide detection techniques are essential to monitor and regulate pesticide levels in soil and water, ensuring compliance with safety standards, minimizing environmental impact, and safeguarding society from potential health hazards.^12^

One of the most used pesticides is 2,4-dichlorophenoxyacetic acid (2,4-D), which holds considerable importance in agriculture due to its low cost and high selectivity.^13^ However, the significant agricultural reliance on 2,4-D necessitates a comprehensive understanding of its environmental fate. Residues of this herbicide can impact ecosystems since its high mobility and persistence in an aqueous medium have caused serious concern.^14,15^ Toxicological studies indicate that long-term exposure to 2,4-D can cause many problems such as the mortality of fish eggs, causing a lower reproduction rate,^16^ and increasing mortality and malformations of amphibians.^17^ This pesticide can even induce problems in human beings, such as a significant increase in the odds of a prostate cancer diagnosis.^18^ Prenatal exposure to 2,4-D was associated with slower auditory signal transmission in early infancy^19^ and a possible association with the increasing risks of non-Hodgkin lymphoma (NHL).^20^ In consequence, the EPA (United States Environmental Protection Agency) establishes in its National Primary Drinking Water Regulation (updated in December 2024) the MCLG (Maximum Contaminant Level Goal) of 0.07 mg/L (70 ppb) for drinking water. According to the EPA, the MCLG is the level of a contaminant in drinking water below which there is no known or expected risk to health, allowing a margin of safety, and is a nonenforceable public health goal.^21^ In this sense, the development of precise detection techniques is essential in advancing sustainable agricultural practices and mitigating the negative consequences associated with 2,4-D application.

Surface-enhanced Raman scattering (SERS) emerges as a powerful, sensitive technique for contaminant detection.^22^ SERS operates on the principle of enhancing Raman signals through the interaction of the target molecules with nanostructured metallic surfaces, typically composed of copper, gold, and silver for excitation lasers in the visible.^23,24^ This enhancement phenomenon allows for the detection of molecular vibrations at extremely low concentrations, making SERS an alternative for contaminant detection. The high sensitivity of this technique enables the identification and quantification of trace amounts of molecules, including pesticides,^25,26^ pharmaceuticals,^27,28^ and biomolecules.^29,30^

The localized surface plasmon resonance (LSPR) produces a huge enhancement of the electromagnetic field on these nanoparticles, which is highly dependent on their size, shape, aggregation, and surface composition. Therefore, the choice of the type of nanoparticles in SERS is important due to their determinant influence on the efficacy and sensitivity of the final detection, since the use of appropriate nanoparticles will lead to a high Raman response of molecules of interest adsorbed on the metal nanoparticles.^31–33^

Nevertheless, to obtain the signal of molecules that are difficult to detect, some theoretical approaches can be applied, such as machine learning in the data analysis to detect a reasonable signal of the analyte.^34,35^ On the other hand, experimental approaches using nanoparticles with more intricate structures are employed.^36–42^ For this purpose, many steps and long syntheses are necessary, in addition to the use of grafting agents or different substrates for the deposition of plasmonic nanoparticles. Table 1 shows some intricate nanoparticles and the analysis conditions used for the detection of pesticide 2,4-D.

**Table 1:** Nanoparticles and Analysis Conditions for the Detection of the Pesticide 2,4-D in Different Matrices

nanoparticle	size	laser	sample	LOD (mol/L)	references
Au@Ag	21.25@2.31	785	tea	1.88 × 10^–10^	^36^
HAu@AgNFs@MBA	-^a^	785	tea and milk	4.98 × 10^–10^	^37^
AuNPs@Silica	4.62	785	milk	4.52 × 10^–11^	^38^
TiO_2_NTAs@5 nm-AuNPs	5	633	water	3.2 × 10^–11^	^39^
AuNP@MSF	5.15	785	food	3.57 × 10^–11^	^40^
paste-collect test with AgNPs	78	638	tomatoes	3.63 × 10^–10^	^41^
AgNPs-gum Arabic electrochemically codeposited	50–70	514	water	1.0 × 10^–12^	^42^

a Not mentioned.

In this work, we pursued 2,4-D detection by using the simplest nanoparticles possible, avoiding complex and long synthesis methods. One of the goals of this work was the morphological modification of Ag nanoparticles by modifying the amount of citrate added to the mixture. Citrate has two different roles in the Ag NPs synthesis: (a) chemical reducer of Ag^+^ ions and (b) stabilizer of the resulting NPs due to its adsorption on the interface and the negative potential induced by this adsorption because of the negative charge provided by the ionized citrate at neutral pH. These two roles are highly connected since the adsorption, while the nanoparticle is growing, can modulate the morphology of the resulting nanoparticle. Regarding the latter fact, the concentration of citrate is something important in the final SERS performance of Ag NPs, and this is one of the main goals of the present work. In addition, the modification of the electric charge of citrate-capped NPs can be essential in the detection of acidic analytes such as the pesticide 2,4-D. In this sense, the optimization of the synthesis of silver/citrate colloid is a crucial step, making the nanoparticles suitable for the detection of this particular contaminant.

## 2 Materials and Methods

### 2.1 Materials

Silver nitrate (AgNO_3_, 169.87 g/mol) was acquired from Merck; 2,4-dichlorophenoxyacetic acid (2,4-D, C_8_H_6_Cl_2_O_3_, 221.04 g/mol, analytical standard), sodium citrate tribasic dihydrate (C_6_H_5_Na_3_O_7_·2H_2_O, 294.10 g/mol, 99.0%), and nitric acid (HNO_3_, 65% Suprapur) were purchased from Sigma-Aldrich. All of the solutions were prepared using ultrapure water with 18.2 MΩ from a Milli-Q system.

### 2.2 Silver Colloid Synthesis

Silver nanoparticles reduced by citrate (AgCit) were obtained according to the method described by Lee and Meisel.^43^ The synthesis was slightly modified in some cases by using different citrate:metal ratios (Figure 1). Briefly, a total of 1.0 mL of trisodium citrate aqueous solution at different concentrations (1.0, 0.50, and 0.25% w/v) was added to 50 mL of a boiling 1.0 × 10^–3^ mol/L silver nitrate aqueous solution, and the boiling was continued for 1 h in the absence of light. The obtained colloids (AgCit_1.0_, AgCit_0.50_, and AgCit_0.25_) showed a turbid gray color and a final pH of 5.5.

**Figure 1:**
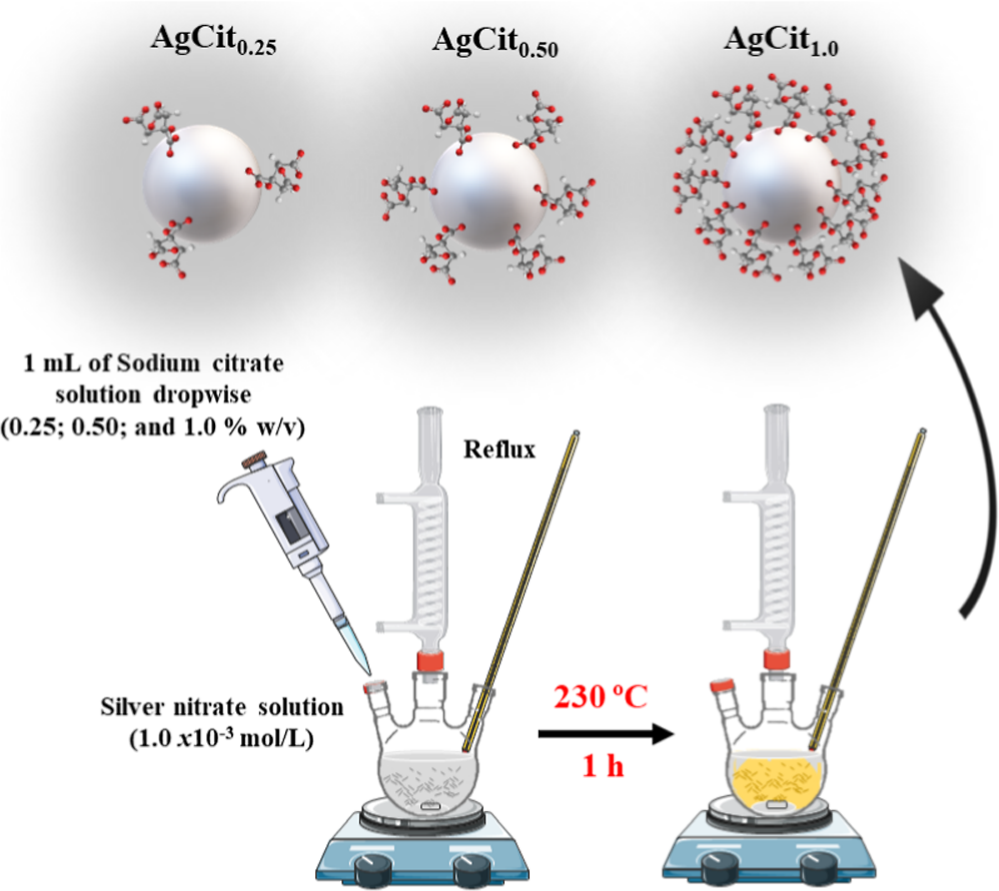
Scheme of the synthesis of different silver nanoparticles by varying the citrate concentration (0.25, 0.50, and 1.0% w/v).

### 2.3 Characterization of Colloids

The UV–vis absorption spectrometer (Shimadzu 3600 UV–vis absorption spectrometer) was employed to obtain the extinction or absorption spectra of AgCit nanoparticles in the presence and absence of 2,4-D. The colloidal suspension for UV–vis absorption was prepared by adding 500 to 2500 μL of ultrapure water. Transmission electron microscopy (TEM) images were obtained by transmission electron microscopy (TEM) using a JEM1400 plus microscope. The samples for TEM were prepared by depositing a drop of the colloidal suspension on grids for TEM (carbon film on 200 mesh copper). The particle size was obtained from ImageJ software, and the normal distribution was plotted. Dynamic light scattering (DLS) and zeta potential measurements were performed using a Zetasizer Nano analyzer (Malvern, ZS90), using an angle of 90° and a source of λ = 633 nm.

### 2.4 Preparation of Samples for SERS Measurements

The SERS samples with no modification of pH were prepared by adding 500 μL of colloids “as-prepared”. Samples prepared at pH 2.0 were prepared by adding 50 μL of 0.1 mol/L HNO_3_ to the mixture. For the samples containing the pesticide, a calculated volume from a stock solution of 2,4-D (1 × 10^–3^ mol/L in water) was added to the mixture. Finally, 500 μL of the final suspension was placed in a cylindrical cuvette of 5 mm, and the laser was focused into the solution to obtain the SERS spectrum.

### 2.5 Raman and SERS Measurements

Raman and SERS spectra were obtained by a micro-Raman inVia Renishaw spectrometer (Wotton-Under-Edge, Gloucestershire, UK), equipped with an electrically cooled CCD camera and a Leica DM 2500 microscope. The laser at 532 nm (Nd/YAG) was employed as the excitation source and a diffraction grating with 1800 L/mm. The Raman signal was collected over the range of 100–4000 cm^–1^. All of the spectra were recorded with 10 s of integration time, 2 accumulations, an objective lens of 50×, and using glass vials. Quantitative analyses were performed by SERS; for this purpose, all SERS and Raman analyses were performed in triplicate, and the standard deviation values were used in the limit of detection calculations, as can be seen in the Supporting Information.

## 3 Results and Discussion

The colloidal metallic suspensions presented the characteristic gray color of silver nanoparticle colloids reduced by sodium citrate. After synthesis, the colloids turned yellowish-brown. Figure 2 shows the extinction spectra by UV–vis spectroscopy, with the plasmon peaks at 405, 414, and 417 nm for AgCit_1.0_ (reduced with 1.0% citrate solution), AgCit_0.50_ (reduced with 0.50% citrate), and AgCit_0.25_ (reduced with 0.25% citrate), respectively. These maxima indicate that the average size of the nanoparticles integrating these colloids is similar but increases slightly in the order AgCit_1.0_ < AgCit_0.50_ < AgCit_0.25_. As previously discussed in the literature, the silver nanoparticles reduced by the method proposed by Lee and Meisel present a wide dispersity of size and shape, which is responsible for the broad surface plasmon absorption with a maximum of around 420 nm.^44,45^

**Figure 2:**
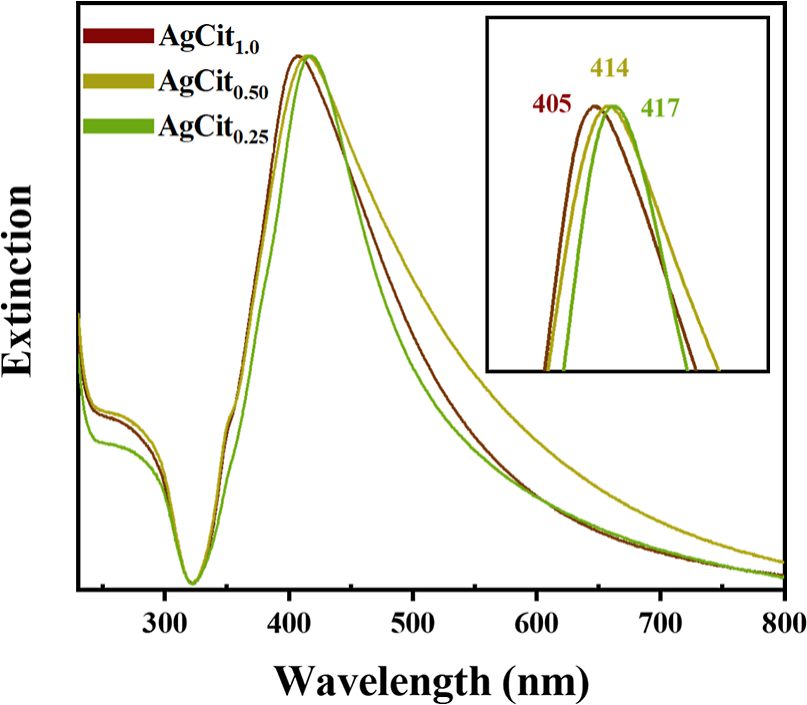
Normalized extinction spectra of silver colloids produced with different concentrations of citrate (1.0, 0.50, and 0.25% w/v) at pH 5.5.

It was reported in the literature that silver nanoparticles prepared by the citrate reduction method produce relatively large sizes and dispersity of the particle shape.^44,46^ In our case, TEM images of the resulting suspensions (Figure 3) confirmed that the broad bands displayed in the extinction spectrum are caused by a relatively wide dispersion of shapes and sizes. However, no huge differences can be seen in the morphology of the different NPs prepared at varying citrate concentrations. For instance, the AgCit_0.25_ sample is integrated by relatively smaller nanoparticles (69.86 ± 19.19 nm). The AgCit_0.50_ sample contains nanoparticles showing a larger size distribution with diameter nanoparticles slightly larger than AgCit_0.25_, achieving an average of 74.27 ± 22.81 nm. However, the images of the colloid AgCit_1.0_ demonstrated the formation of some long and thin needles, and the distribution of sizes and shapes for the nonelongated NPs is similar to that of the AgCit_0.50_ NPs, displaying nanoparticles with 73.17 ± 20.87 nm of average diameter.

**Figure 3:**
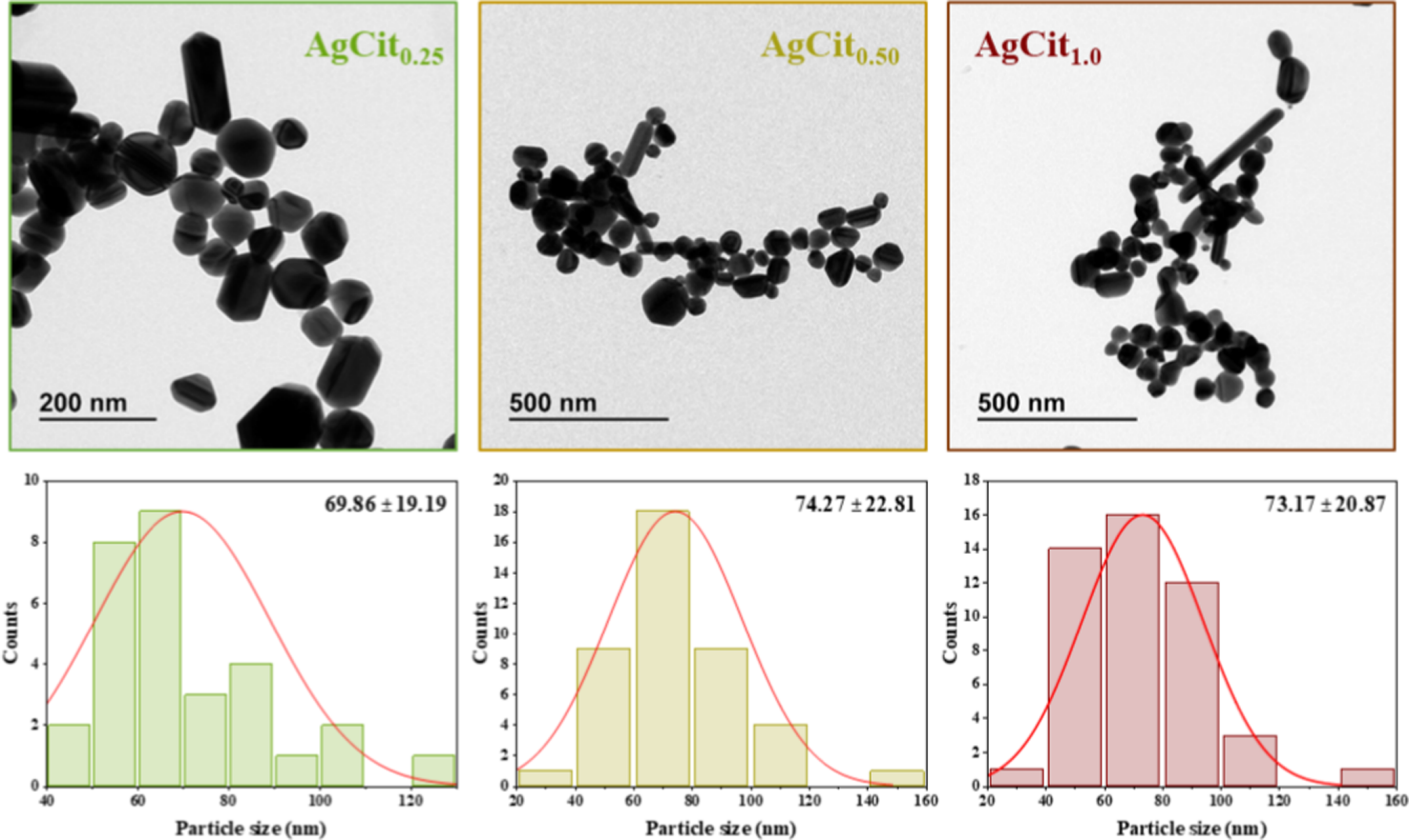
TEM images of AgCit_*x*_ synthesized and the histogram with normal distribution and the average of particle size showing a different size for the nanoparticles depending on the concentration of citrate.

Similar to what is described in the literature, silver-citrate nanoparticles are known by their polydispersity.^44,46^ Here, the nanoparticles present a morphological variation, depending on the citrate amount. As well, the surface charge varies according to the citrate content on the silver surface, with the zeta potential values ranging from −29.8 (±3.1), −32.4 (±3.8), and −39.1 (±3.4) mV for colloids from AgCit_0.25_ to AgCit_1.0_, respectively. The carboxylate groups of citrate and other subproducts of the oxidation are deprotonated at the pH of the aqueous solution, resulting in negatively charged ions (−COO^–^) that interact with the silver nanoparticles.^47,48^ In this sense, the negative charge on the surface of AgCit_*x*_ nanoparticles helps to prevent their aggregation by providing electrostatic repulsion between other nanoparticles, contributing to the stability of the nanoparticle dispersion.^49^ As expected, with the citrate content in the reduction process increasing, the zeta potential decreases due to the presence of a greater number of carboxylate groups.

The SERS analysis strongly depends on the possibility of adsorption of the analyte onto the plasmonic NPs, giving rise to Raman enhancement. Therefore, the key point in the detection of 2,4-D is to find those experimental conditions that facilitate its interaction with the surface. The adsorption process of 2,4-D molecules on a given surface depends on the pH of the medium in which the analyte is found because of the importance of electrostatic forces in the adsorption process. On the other hand, the electric charge of a certain acidic analyte, such as 2,4-D, can be modified by changing the pH. The p*K*_a_ of the pesticide is considered to be between 2.4 and 2.8 (Figure 4).^50–52^ This means that at pH > p*K*_a_, almost all 2,4-D molecules are present in the anionic form, and at pH < p*K*_a_, the 2,4-D species predominates in the neutral form. Therefore, at pH above the p*K*_a_, most of the molecules stay in the aqueous bulk due to the extensive hydration with water molecules. In contrast, at pH below the p*K*_a_, the most abundant neutral form tends to interact with the interface, thus increasing the amount of 2,4-D molecules on the surface. The adsorption mechanism can then proceed via van der Waals forces^51^ or via the formation of a metallic complex with the carboxylate.^53^

**Figure 4:**
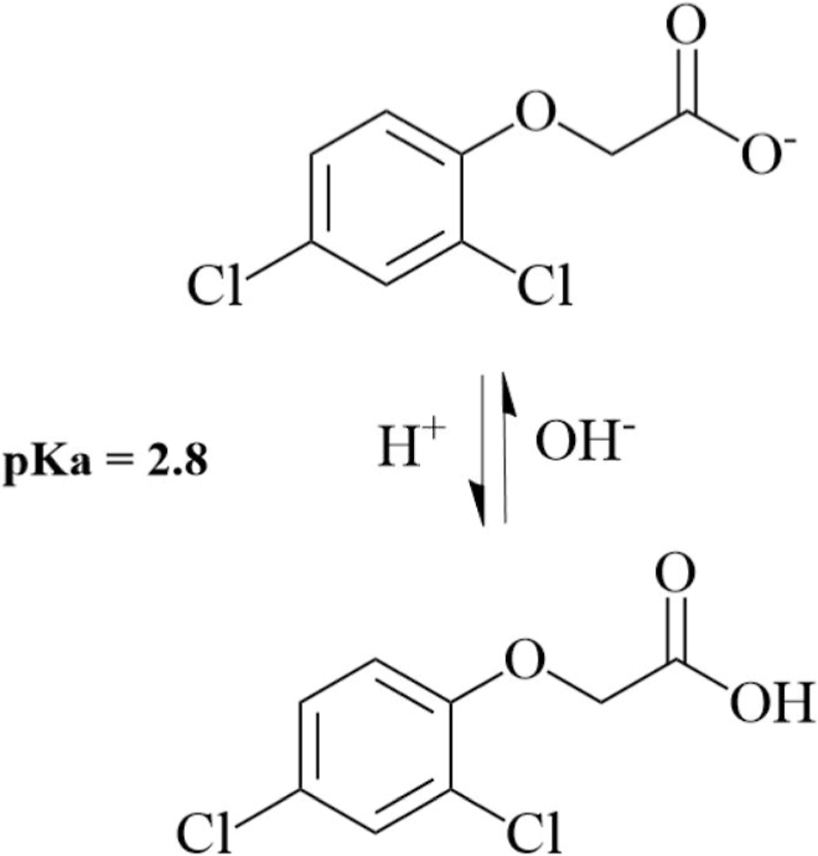
2,4-D molecule protonation in different pH can present the protonated (below the p*K*_a_ value) and deprotonated forms (above the p*K*_a_ value).

For this reason, we have reduced the pH of the resulting Ag colloids in an attempt to stimulate the 2,4-D adsorption on silver. UV–vis spectra reveal the presence of AgCit_*x*_ colloids (pH 2) in the absence and presence of the investigated pesticide, as depicted in Figure 5. In an acidic medium (Figure 5a), only the AgCit_1.0_ colloid exhibits a distinct displacement of a second band, surpassing the other colloids and extending toward the near-infrared (NIR) region due to the aggregation of the nanoparticles upon the decrease of pH, due to the adsorption of H^+^ ions, which may balance the residual negative charge of the surface provided by citrate. In the presence of the 2,4-D pesticide, all of the colloids showed the formation of a second band. In the case of the AgCit_0.50_ and AgCit_0.25_ (Figure 5c,d, respectively) suspensions, the aggregation band was observed at a concentration of 1.0 × 10^–5^ mol/L or higher. However, in the case of the AgCit_1.0_ colloid (Figure 5b), the second aggregation band was observed only at the highest concentration of the pesticide (1.0 × 10^–4^ mol/L). This is attributed to the extensive adsorption of citrate ions on the surface at pH 2, which highly hinders the aggregation of nanoparticles.

**Figure 5:**
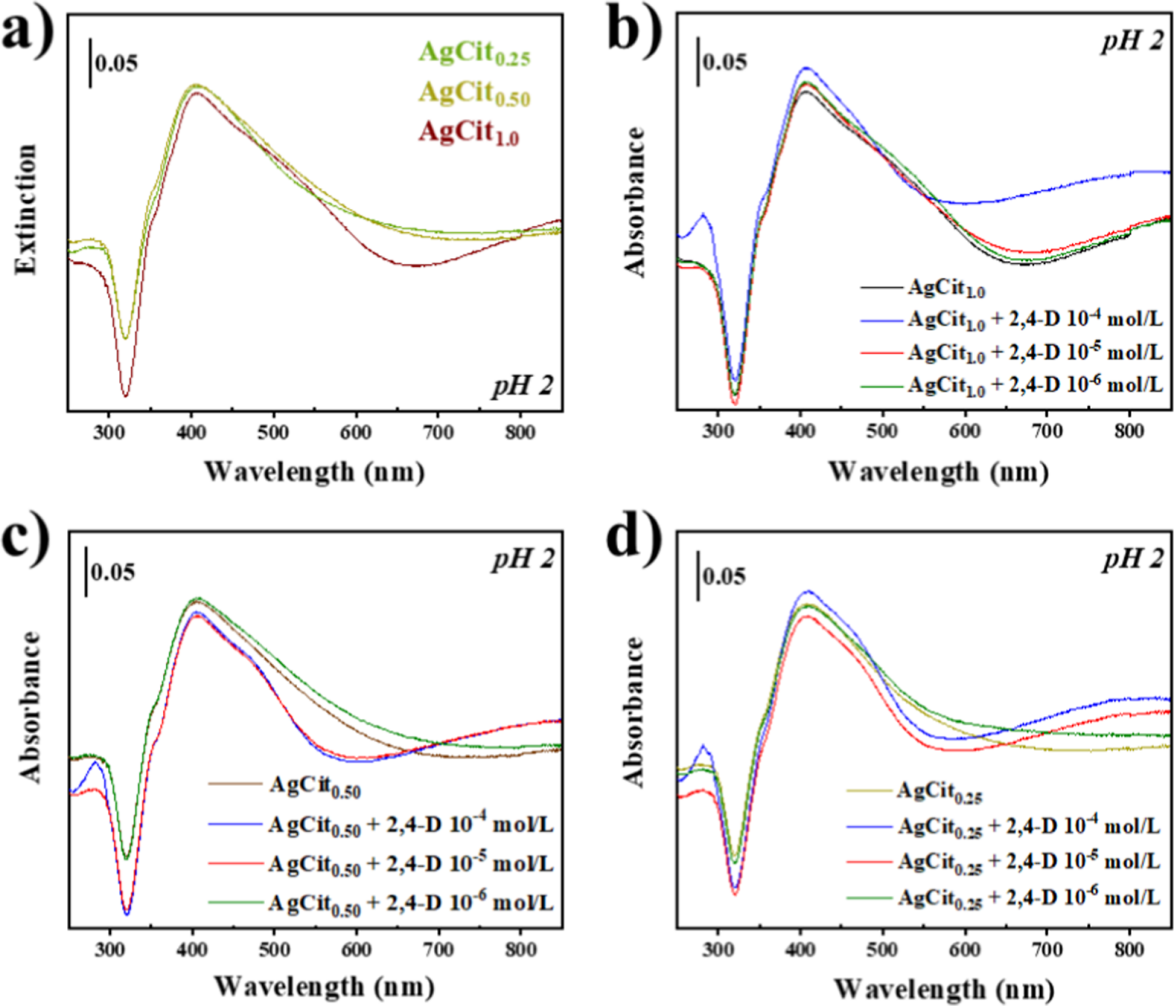
(a) UV–vis spectra of AgCit_*x*_ at pH 2; (b) UV–vis spectra of AgCit_1.0_ at pH 2 + 2,4-D; (c) UV–vis spectra of AgCit_0.50_ at pH 2 + 2,4-D; (d) UV–vis spectra of AgCit_0.25_ at pH 2 + 2,4-D.

As a result of the variation in the pH of the medium, the SERS spectra of the silver-citrate nanoparticle colloids also present a profile different from that of the as-prepared AgCit_*x*_. Figure 6 shows the Raman spectra of AgCit_*x*_ at pH 2 (lower than the p*K*_a_ of 2,4-D) and at the initial pH of the suspension (∼5.5). No AgCit_*x*_ colloids with this initial pH present visible peaks exciting at 532 nm. In contrast, at pH 2, strong peaks attributed to citrate appear at 950 cm^–1^ υ_s_(C–C) and (C–COO), 1024 cm^–1^ υ_s_(COO−), and 1393 cm^–1^ υ_as_(COO−).^54^ The assignment of the main citrate peaks shown in the SERS spectra is presented in Table S3 and in Supporting Information. Nevertheless, the spectra of AgCit_0.25_ and AgCit_0.50_ present smaller citrate signals in comparison to those of the AgCit_1.0_ colloid. The analysis of the above citrate bands indicates that the carboxylate groups are attached to the surface and that they adopt a perpendicular orientation regarding the metal surface.^54^ Furthermore, a band at 232 cm^–1^ was always observed in the SERS spectra of all colloids. This band is attributed to the Ag-citrate interaction by the COO–Ag bond.^54^

**Figure 6:**
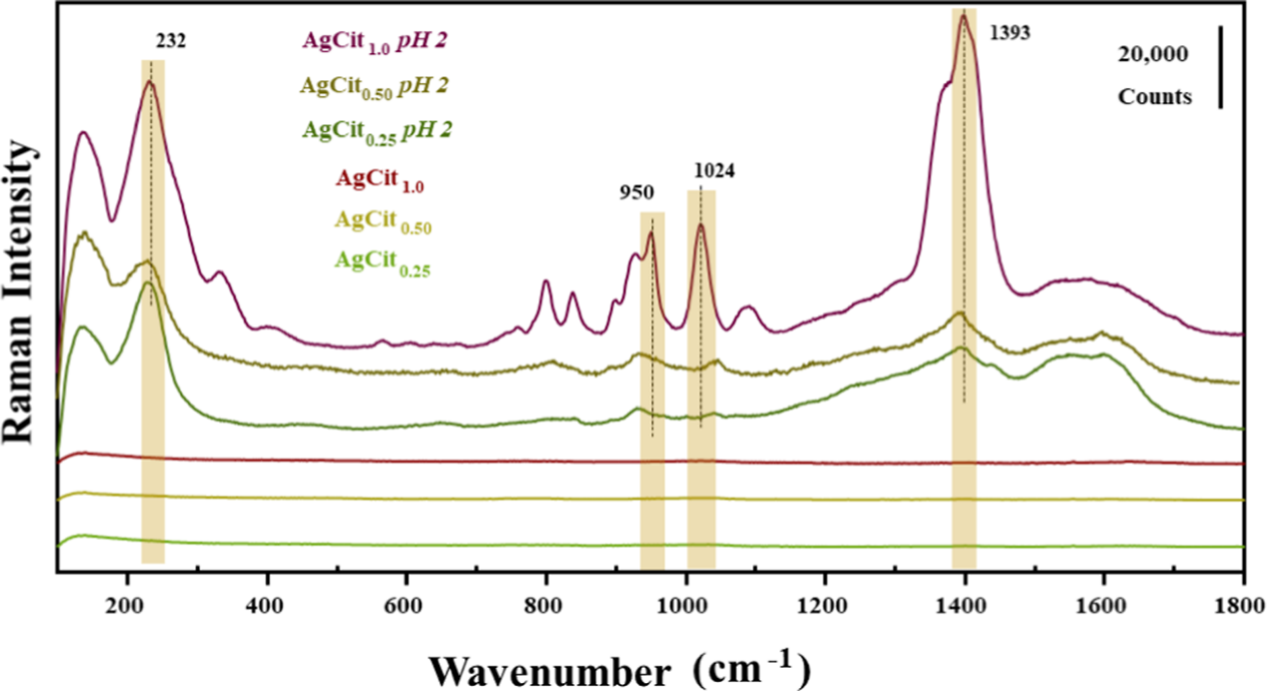
Raman spectra of AgCit_*x*_ nanoparticles at pH 2 and as-prepared (pH 5.5). Highlighted are the main citrate peaks. All spectra were obtained using LL = 532 nm, 10 s, two accumulations, and laser power of 100%.

The high intensity of citrate signals observed for AgCit_1.0_ becomes undesirable for detecting molecules with difficult-to-obtain signals such as 2,4-D since they can overcome the weak signal from the analyte. For this reason, we did a reduction of Ag^+^ by using a lower amount of citrate in order to reduce the contribution from the citrate bands.

Figure 7 displays the Raman spectra of the high-concentration solutions of 2,4-D (1.0 × 10^–3^ mol/L), both at the initial pH of the suspension (pH ∼ 5.5) and at acidic pH 2, compared with the pesticide in the solid state. As can be seen, no bands corresponding to 2,4-D were observed at this high concentration. The highlighted peaks (393 and 1593 cm^–1^) correspond to those observed in the SERS spectra when in contact with the proposed colloids, which refer to the δ­(CCl) and ν_as_(COO^–^), respectively.^55^ The complete assignment of the 2,4-D powder peaks is presented in Table S2 in the Supporting Information.

**Figure 7:**
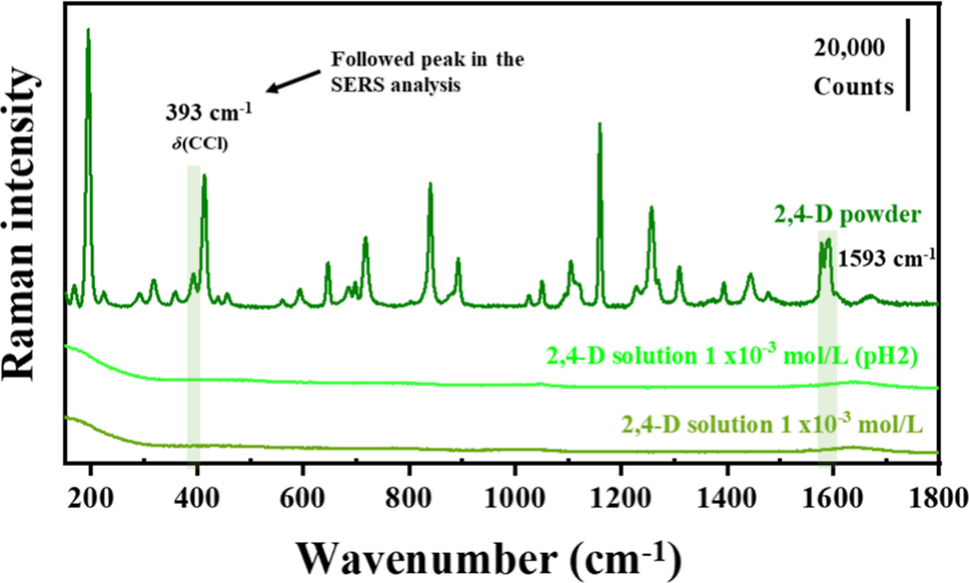
Raman spectra of 2,4-D in powder and solutions at 1.0 × 10^–3^ mol/L at the initial pH (∼4.5) and pH 2.0. Highlighted are the peaks of 2,4-D that clearly appear in the SERS spectra. All spectra were obtained using LL = 532 nm, 10 s, two accumulations, and laser power of 100% for the solutions and 50% for the powder.

The SERS of 2,4-D was proven by us at different conditions (AuNPs, AgNPs, magnetite/AuNPs, cellulose/AuNPs, and paper + NPs), laser lines (514, 633, 785, and 852 nm), solution pH (2–10), pesticide concentration (from 1 × 10^–3^ to 1 × 10^–8^ mol/L), and type of sample (solid substrate and colloidal suspension), as can be observed in Table S1 (Supporting Information). All these previous experiments did not provide good results on the detection of the pesticide. In fact, no SERS bands could be obtained. As previously mentioned, the reason for this is that 2,4-D is poorly adsorbed on the surface of Ag NPs due to its negative charge at the initial pH of the AgNP suspension (5.5). Also, the relatively high concentration of citrate in conventional Ag-citrate colloids could compete with 2,4-D in the adsorption on the Ag surface.

For the above reasons, the pH was reduced below the p*K*_a_ of 2,4-D in an attempt to stimulate the adsorption of 2,4-D. Figure 8 shows the SERS spectra obtained for colloids of AgCit_1.0_ at different 2,4-D concentrations (ranging from 1.0 × 10^–4^ to 1.0 × 10^–6^ mol/L) and reducing the pH to 2.0. At these conditions, the SERS bands of the pesticide are only observed at concentrations above 1.0 × 10^–5^ mol/L, with bands of 393 cm^–1^ shifting to 397 cm^–1^ in the SERS spectra, corresponding to the C–Cl vibrations of the pesticide.^41,56^ At concentrations below the latter critical concentration, the spectrum is dominated by citrate bands. The band at 1397 cm^–1^, related to the υ_as_(COO^–^) vibration, appears with high intensity in the AgCit_1.0_ colloid. The assignment of citrate peaks shown in the SERS spectra is presented in Table S3 in the Supporting Information.

**Figure 8:**
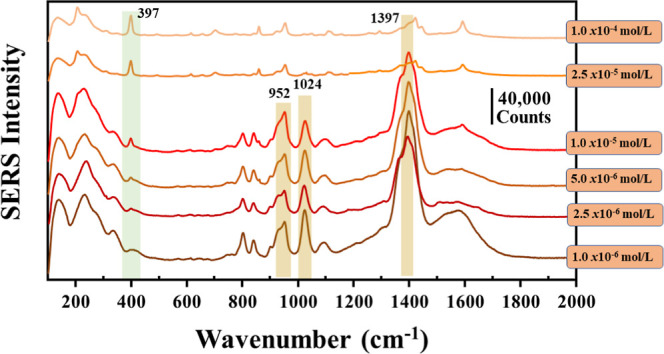
SERS spectra of AgCit_1.0_ + 2,4-D (pH 2) at final contaminant concentrations from 1.0 × 10^–4^ to 1.0 × 10^–6^ mol/L. Highlighted in green is the peak of 2,4-D and in orange are the main peaks of citrate. All spectra were obtained using LL = 532 nm, 10 s, two accumulations, and laser power of 100%.

The large contribution of citrate bands on the SERS spectrum led us to try the fabrication of AgNPs at lower citrate concentrations. Figure 9 shows the SERS spectra obtained for colloids AgCit_0.50_. In this case, clear bands corresponding to 2,4-D can be seen at lower concentrations than in the case of the AgCit_1.0_ colloid (1.0 × 10^–4^ to 5.0 × 10^–8^ mol/L). Although citrate bands are still seen at low concentrations, mainly below 2.5 × 10^–5^, no overlapping is seen as in the case of the AgCit_1.0_ colloid.

**Figure 9:**
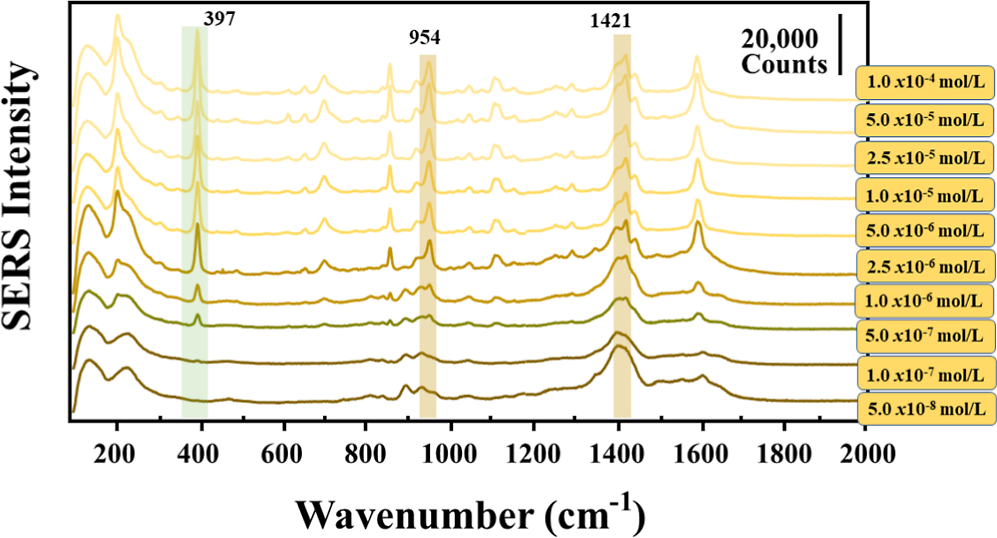
SERS spectra of AgCit_0.50_ + 2,4-D (pH 2) at final contaminant concentrations from 1.0 × 10^–4^ to 5.0 × 10^–8^ mol/L. Highlighted in green is the peak of 2,4-D and in orange are the main peaks of citrate. All spectra were obtained using LL = 532 nm, 10 s, two accumulations, and laser power of 100%.

SERS spectra on the AgCit_0.50_ colloid show clear differences in comparison to the Raman spectrum of the solid 2,4-D. The appearance of new peaks at 1397 and 957 cm^–1^, attributed to υ_s_(COO−) and υ­(C–COO−), reveals that the pesticide is under the ionized carboxylate form on the Ag surface. In addition, the bands at 1160 and 1258 cm^–1^ almost disappear, while a prominent band is now seen at 1108 cm^–1^. Prominent bands appear at ca. 200 and 397 cm^–1^ in the SERS spectra assigned υ­(C–Cl). Finally, the υ­(C=C) stretching band of the aromatic ring at 1600 cm^–1^ is enhanced. All of these spectral changes suggest the interaction of 2,4-D with the Ag surface via the complexation with the carboxylate moiety through a perpendicular orientation with respect to the surface.

Figure 10 shows the spectra obtained for colloids AgCit_0.25_ with pesticide concentrations from 1.0 × 10^–4^ to 1.0 × 10^–7^ mol/L. In this case, citrate bands are practically nonexistent. The use of a much lower citrate concentration leads to an even lower amount of citrate anions on the surface of the AgCit_0.25_ colloid. Thus, facilitating the interaction between the 2,4-D pesticide and silver. Furthermore, the higher polydispersity found in AgCit_0.25_ did not demonstrate any negative impact on the detection of 2,4-D, since the difference compared to that of other colloids is small.

**Figure 10:**
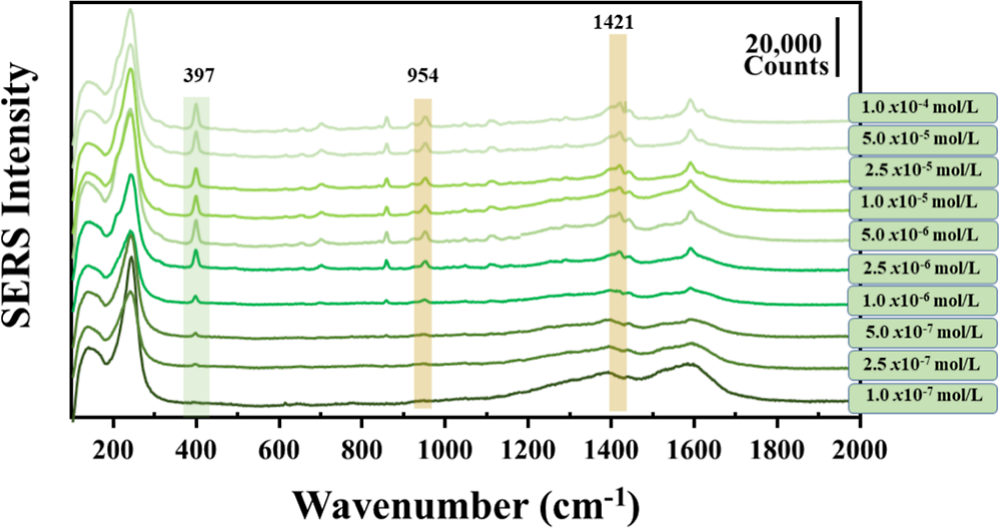
SERS spectra of AgCit_0.25_ + 2,4-D (pH 2) at final contaminant concentrations from 1.0 × 10^–4^ to 1.0 × 10^–7^ mol/L. Highlighted in green is the peak of 2,4-D and in orange are the main peaks of citrate. All spectra were obtained using LL = 532 nm, 10 s, two accumulations, and laser power of 100%.

Therefore, the use of a lower citrate concentration in the reduction process of Ag^+^ ions leads to a lower amount of citrate anions on the surface of the AgCit_0.50_ and AgCit_0.25_ colloids, facilitating the interaction between the 2,4-D pesticide and silver and finally leading to a notable increase in signal intensity due to the SERS effect.

### 3.1 Quantitative Analysis

The ratio of the area of the 2,4-D band at 397 cm^–1^ and that of the water band at 3400 cm^–1^ (2,4-D_397_/Water_3400_) was used to plot the variation of the SERS intensity against the pesticide concentration,^26^ as also reported in previous analysis for other pesticides.^55^ Figure 11 shows the variation of the latter ratio for the different pesticide concentrations on the three different employed colloids, always maintaining the pH at 2.0.

**Figure 11:**
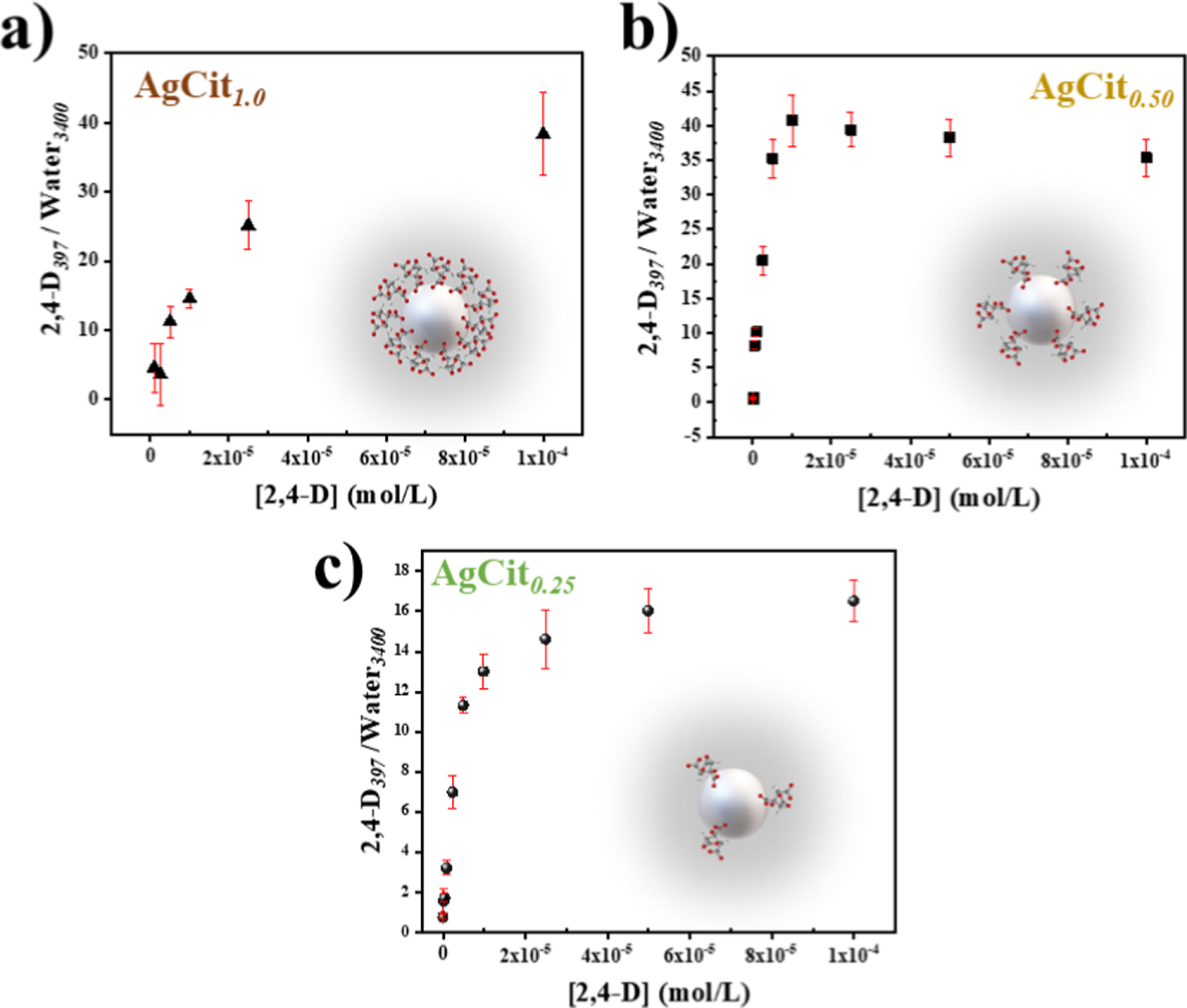
Plot of the ratio of the band area at 397 cm^–1^ from 2,4-D and the band area from water at 3400 cm^–1^ for (a) AgCit_1.0_, (b) AgCit_0.50_, and (c) AgCit_0.25_, respectively.

The plots of the SERS intensity displayed in Figure 11 can be used to further analyze the adsorption mechanism of 2,4-D on the surface. According to Giles et al.,^57,58^ the adsorption can occur through different mechanisms. AgCit_1.0_ presents an L-curve profile (Figure 11a), which describes an increasing adsorption in which the pesticide does not clearly show a limited sorption capacity on the nanoparticle due to the difficulty of finding the vacant sites covered by the citrate molecules on the silver surface, without finding a well-defined sorption plateau. On the other hand, the silver nanoparticles reduced by lower citrate concentrations (AgCit_0.5_ and AgCit_0.25_) present a traditional Langmuir isotherm (Figure 11b,c), describing a high affinity between silver nanoparticles and the pesticide in a monolayer, providing an adsorption value for the lower concentrations and achieving such a stable plateau. The isotherm definition helps to explain the higher efficiency of the lower citrate concentrations (AgCit_0.50_ and AgCit_0.25_) when compared to AgCit_1.0_. In this sense, a threshold for the detectability of 2,4-D is observed (Table 2). For this pesticide, the traditional silver-citrate nanoparticle (AgCit_1.0_) does not present a regular adsorption profile in comparison with that of the silver-citrate nanoparticles synthesized with lower citrate concentrations (AgCit_0.50_ and AgCit_0.25_). This behavior probably is due to the decrease in citrate intensity observed in the SERS spectra of AgCit_0.50_ and AgCit_0.25_ when compared with AgCit_1.0_, indicating a smaller citrate layer coverage, which increases the probability of the pesticide reaching the silver nanoparticle.

**Table 2:** LOD Values Obtained by Different Methods

	AgCit_1.0_	AgCit_0.50_	AgCit_0.25_
LOD by the signal-to-noise approach (mol/L)	3.10 × 10^–6^	1.85 × 10^–7^	1.20 × 10^–7^
LOD by linear regression (mol/L)	1.01 × 10^–6^	3.39 × 10^–8^	1.43 × 10^–7^
*R* ^2^	0.96089	0.95873	0.99681
sensitivity from linear regression	834.98	8876.25	2604.00

This deviation of the AgCit_1.0_ colloid behavior can be explained on the basis of the large amount of citrate existing on its surface. Under these conditions, direct adsorption of 2,4-D on the silver surface is difficult since there can exist other possibilities of adsorption, such as the interaction of 2,4-D with citrate ions covering the Ag NPs. Conversely, on AgCit_0.50_ and AgCit_0.25_ colloids, the lower amount of citrate leaves more free surface, leading to a Langmuir-like adsorption behavior.

The LOD values can be calculated by a signal-to-noise approach. According to Desimoni and Brunetti,^59^ the LOD by S/N ratio should be equal to 3.0, which allows proving the presence of the analyte in the evaluated sample with a probability larger than 99%. By this method, the LOD by S/N was calculated by measuring three different spectra, according to the data presented in Table S4 (see Supporting Information), and the results achieved the LOD of 1.20 × 10^–7^, 1.85 × 10^–7^, and 3.10 × 10^–6^ mol/L for AgCit_0.25_, AgCit_0.50_, and AgCit_1.0_, respectively. To summarize, Table 2 presents the LOD value found by both methods.

In addition, linear regression can be used to calculate the limits of detection (LOD) (see the Supporting Information). All values and data used for LOD calculations by these methods are presented in Table S4. First, linear regression was performed considering the linear region of each Langmuir curve (2.5 × 10^–5^ to 1.0 × 10^–6^ for AgCit_1.0_, 5.0 × 10^–6^ to 5.0 × 10^–8^ for AgCit0_.50_, and 2.5 × 10^–6^ to 1.0 × 10^–7^ AgCit0_.25_). Considering this linear region, LODs of 1.01 × 10^–6^, 3.39 × 10^–8^, and 1.43 × 10^–7^ mol/L were found for AgCit_1.0_, AgCit_0.50_, and AgCit_0.25_, respectively. The sensitivity of the linear regressions was determined by the slope of the analyzed points, as shown in the Supporting Information. This sensitivity achieved higher values for the AgCit_0.50_ and AgCit_0.25_ colloids, indicating greater linearity in the dependence between the concentration and signal. This finding also demonstrates that there is a threshold where the optimal conditions were achieved with citrate concentrations less than 1.0.

Considering the EPA limit of 0.07 mg/L (MCLG) for 2,4-D in drinking water, when converted to molar concentration, the established limit is 3.17 × 10^–7^ mol/L. Analyzing the values obtained using both LOD calculations (by the signal-to-noise approach and by linear regression), the AgCit_0.50_ and AgCit_0.25_ colloids demonstrated the ability to reach the order of 10^–7^ mol/L, nearby the regulatory limit. However, the AgCit_1.0_ colloid is capable of detecting only concentrations above the established limit (10^–6^ mol/L). Herein, it is possible to observe a cutoff range in the LOD values, where the traditional silver-citrate colloid (AgCit_1.0_) presents a lower LOD value, no matter the employed LOD calculation method. Therefore, the use of lower amounts of the reducing agent (AgCit_0.50_ and AgCit_0.25_) is highly advantageous for the SERS efficiency in the detection of 2,4-D. Indeed, this efficiency is enhanced at a pH lower than the p*K*_a_ of the herbicide. All of these effects are attributed to the presence of citrate. On one hand, a lower amount of citrate is better to avoid any hindrance effect on the adsorption of 2,4-D. On the other hand, at low pH, citrate exhibits a potentiated hindrance effect due to the stimulated adsorption upon reduction of the affinity with the aqueous medium. Thus, a reduction in the amount of citrate leads to better SERS results. Finally, the better results obtained by using the AgCit_0.50_ colloid regarding the AgCit_0.25_ one are attributed to the lower total available surface for the adsorption exhibited by the latter colloid on the basis of the bigger size of its Ag NPs. This constrains the total number of 2,4-D adsorbed on the available surface, leading to a lower SERS signal.

## 4 Conclusion

In conclusion, this study demonstrates the effectiveness of surface-enhanced Raman scattering (SERS) as a powerful analytical technique for the detection of pesticides, particularly 2,4-dichlorophenoxyacetic acid (2,4-D). Additionally, this investigation into the synthesis of silver nanoparticles has achieved valuable insights into the role of citrate concentration in optimizing the SERS signal intensity. By the modified synthesis parameters, the results demonstrated the ability to improve the properties of silver nanoparticles for enhanced detection of pesticides, reaching LOD values from 3.10 × 10^–6^ (AgCit_1.0_) to 1.85 × 10^–7^ (AgCit_0.50_) and 1.20 × 10^–7^ mol/L (AgCit_0.25_) by the signal-to-noise ratio approach (S/N ratio). Linear regression corroborates the S/N results, showing lower LOD values for the traditional AgCit nanoparticle (AgCit_1.0_ = 1.01 × 10^–6^, AgCit_0.50_ = 3.39 × 10^–8^, and AgCit_0.25_ = 1.43 × 10^–7^). Based on these findings, it is shown that there is a cutoff range for silver-citrate nanoparticles (AgCit_*x*_), where with reduced concentrations of citrate in the synthesis medium (0.5 and 0.25%), a higher LOD value is found for 2,4-D when compared with the traditional silver-citrate nanoparticles synthesis, being the signal on the AgCit_0.25_ colloid lower due to the limited available surface. These colloids with lower citrate concentrations are capable of detecting concentrations very close to the limit established by the EPA. This behavior is due to the decrease in citrate intensity observed in the SERS spectra combined with the greater probability of the pesticide reaching the silver nanoparticle due to the decrease in the extent of the citrate layer coverage. Overall, the findings underscore the importance of SERS as a versatile tool for environmental monitoring and highlight the potential of silver nanoparticles in advancing the field of molecular detection.

## Supplementary Material



## Notes

Parameters that failed to detect the 2,4-D pesticide; peak attribution for 2,4-D powder; peak attribution for citrate; sodium citrate molecule; parameters obtained from the signal and noise of the colloid + pesticide and neat colloid; and parameters obtained from the linear regression and used in the LOD calculations (PDF)
