# Supplementary material for: Overcoming Detection Challenges of 2,4‑D Herbicides via SERS through a Simple Modification of Citrate-Reduced Silver Nanoparticles
Source: ACS Omega. 2025 Aug 5;10(32):36015–24. doi: 10.1021/acsomega.5c03570 (PMC12368817; doi:10.1021/acsomega.5c03570)
Supplement: Supplementary file 1 [file ao5c03570_si_001.pdf]

Overcoming Detection Challenges of 2,4-D Herbicide via SERS through a Simple Modification of Citrate-Reduced Silver Nanoparticles

Guilherme Dognani[1]*, Francisca Belen Fuenzalida[2], Carlos José Leopoldo Constantino[1], Santiago Sanchez-Cortes[3]*

[1]São Paulo State University (UNESP), School of Science and Technology, 19060-900, Presidente Prudente, SP, Brazil.

[2]Pavol Jozef Safarik University, Faculty of Science, 040 01 Kosice, Slovakia.

[3]Instituto de Estructura de la Materia (IEM-CSIC), E-28006, Madrid, Spain.

* Corresponding author: *guilherme.dognani@unesp.br*

Supplementary Material

1. Previous tests to detect the 2,4-D pesticide

Table S1. Parameters that failed to detect the 2,4-D pesticide.

SERS Substrate	Evaluated parameters	Laser line	Results found
Cellulose/AuNPs (in solid, deposited by *casting*)	Concentration variation (10^{-4} – 10^{-8} mol/L)	*Micro-Raman, 633 and 785 nm	Small or no reference to the pesticide analyzed.
Cellulose/AgNPs (in solid, deposited by *casting*)	Concentration variation (10^{-4} – 10^{-8} mol/L)	*Micro-Raman, 633 nm	Small or no reference to the pesticide analyzed.
Cellulose/AuNPs (in suspension)	pH (2-10), concentration and solvent variation	*Micro-Raman, 514, 633 and 785 nm	Small or no reference to the pesticide analyzed.
Cellulose/AuNPs (in suspension)	Concentration variation (10^{-4} – 10^{-8} mol/L)	**Portable Raman (785 and 852 nm)	Small or no reference to the pesticide analyzed.
Cellulose/AuNPs + 4-aminothiophenol (in suspension)	Concentration variation (10^{-4} – 10^{-8} mol/L)	**Portable Raman (785 and 852 nm)	Slight widening of the band (1072 cm^{-1}).

AgNPs (hydroxylamine)	pH (2-12) and Concentration variation (10^{-4} – 10^{-8} mol/L)	*Micro-Raman, 633 nm	Small or no reference to the pesticide analyzed.
Gold nanorods (AuNRs)	Contact time (5 – 60 min)	*Micro-Raman, 633 nm	Small or no reference to the pesticide analyzed.
Paper substrate + AuNRs	pH 4.5 and 12	*Micro-Raman, 633 and 785 nm	Small or no reference to the pesticide analyzed.
Paper substrate + AgNPs (hydroxylamine)	pH 4.5 and 12	*Micro-Raman, 633 and 785 nm	Small or no reference to the pesticide analyzed.
Paper substrate + AuNPs (sodium citrate)	Concentration variation (10^{-4} – 10^{-8} mol/L)	*Micro-Raman, 633 and 785 nm	Small or no reference to the pesticide analyzed.
PVD silver substrate	Concentration of 10^{-3} mol/L	*Micro-Raman, 785 nm	Small or no reference to the pesticide analyzed.
Magnetite/AuNPs	Concentration of 10^{-3} mol/L	**Portable Raman (785 and 852 nm)	Small or no reference to the pesticide analyzed.

*Micro-Raman (Renishaw spectrograph, model in Via), equipped with a Leica microscope and CCD detector.
**Portable Raman (Bruker, model BRAVO), with Duo Laser™ system (785 and 852 nm) and SSE™ (fluorescence mitigation).

2. 2,4-D powder peak attribution

Table S2. Peak attribution for 2,4-D powder is presented in Figure 7.

Wavenumber (cm^{-1})	Attribution	Reference
558	δ (COC); δ (CC)	1
594	δ $(CC)_{anel\ fora\ do\ plano}$	1
645	υ $(CC)_{anel}$; υ (CCl)	1
716	δ (COO-)	1, 2
838	δ (CH); υ $(CC)_{anel}$; υ (C=O); υ (CCl)	2
852	υ $(CC)_{anel}$; υ (C=O)	1
895	υ $(C\text{-}COO^-)$	1
1104	υ $(CC)_{anel}$; υ (CCl)	3
1161	δ $(CH)_{anel}$	2, 3
1311	υ $(CC)_{anel}$	1, 2
1444	υ_s (COO^-); υ (CC); ω (CH_2)	2, 3
1594	υ_{as} (COO^-); υ $(CC)_{anel}$	3

3. Citrate main peak attribution for the SERS spectra

Table S3. Peak attribution for citrate is presented in Figure 6.

$AgCit_{1.0}$ Wavenumber (cm^{-1})	$AgCit_{0.5}$ Wavenumber (cm^{-1})	$AgCit_{0.25}$ Wavenumber (cm^{-1})	Attribution	Reference
1397	1421	1421	ν(COO^-)	4,5
1024	-	-	ν(C-O)	4,5
952	954	954	ν(C-COO)	4,5
232	233	232	COO-Ag	5

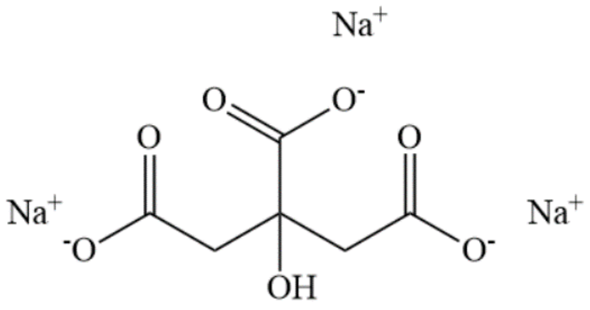

Figure S1. Sodium citrate molecule.

4. Limit of detection (LOD) by Signal-to-noise approach

Table S4. Parameters obtained from signal and noise of colloid+pesticide and neat colloid, respectively.

Colloids noise intensity at 397 cm^{-1}					
**Colloid**	**Measure 1**	**Measure 2**	**Measure 3**	**Average**	**Standard deviation**
**$AgCit_{1.0}$**	164.4184	191.9640	146.2898	167.5574	22.9983
**$AgCit_{0.50}$**	174,8974	147,0278	169,4349	144.3033	32.0434
**$AgCit_{0.25}$**	251.5034	209.5444	169.4349	210.1609	41.0377
**Colloids + pesticide signal intensity at 397 cm^{-1}**					
**Colloid + Pesticide**	**Measure 1**	**Measure 2**	**Measure 3**	**Average**	**Standard deviation**

**$AgCit_{1.0}$ (2.5 x10^{-6} mol/L of 2,4-D)**	402.5687	372.3749	439.9345	404.9594	33.8432
**$AgCit_{0.50}$ (5.0 x10^{-8} mol/L of 2,4-D)**	123.3858	119.4534	107.7544	116.8645	8.1309
**$AgCit_{0.25}$ (1.0 x10^{-7} mol/L of 2,4-D)**	604.1469	525.4236	438.9121	522.8275	82.6480

5. Limit of detection (LOD) by linear regression

The LOD was calculated considering the ratio of the integrated area of the SERS band at 397 cm^{-1} by the area of the water band (3400 cm^{-1}), as follows:

$$LOD = 3x\frac{\sigma}{s}$$

where σ is the standard deviation of the "silver nanoparticle Raman signal" in the absence of the pesticide (noise) and S is the slope of the regression equation (sensitivity). The calculation of the parameters is described in detail below.

Table S5. Parameters obtained from the linear regression and used in the LOD calculations.

	**$AgCit_{1.0}$**	**$AgCit_{0.50}$**	**$AgCit_{0.25}$**
**Linear regression**			
**Fitting equation**	Y = 0.00573 + 834.98229 [2,4-D]	Y = - 6.93938 x10^{-5} + 8876.24662 [2,4-D]	Y = 4.67316 x10^{-4} + 2604.00323 [2,4-D]
**R^2**	0.96089	0.95873	0.99681
**Slope (sensitivity)**	834.98229	8876.24662	2604.00323
**Range of analysis**	2.5 x10^{-5} – 1.0 x10^{-6}	5.0 x10^{-6} – 5.0 x10^{-8}	2.5 x10^{-6} – 1.0 x10^{-7}
**Standard deviation (silver colloids)**	2.804 x10^{-4}	1.002 x10^{-4}	1.239 x10^{-4}

**LOD (mol/L)**	1.01 x10^{-6}	3.39 x10^{-8}	1.43 x10^{-7}

[1] Costa, J.C.S.; Ando, R.A.; Sant'Ana, A.C.; Rossi, L.M.; Santos, P.S.; Temperini, M.L.A.; Corio, P. ***(2009)*** High performance gold nanorods and silver nanocubes in surface-enhanced Raman spectroscopy of pesticides. *Phys. Chem. Chem. Phys*., 11, 7491–7498.

[2] Jia, J.; Jin, X.; Liu, Q.; Liang, W.; Lin, M.; Xu, H. ***(2016)*** Preparation, Characterization and Intracellular Imaging of 2,4-Dichlorophenoxyacetic Acid Conjugated Gold Nanorods. *J. Nanosci. Nanotechnol*. 16: 5, 4936-4942.

[3] Jia, J.; Xu, H.; Zhang, G.; Hu, Z.; Xu, B. ***(2012)*** High quality gold nanorods and nanospheres for surface-enhanced Raman scattering detection of 2,4-dichlorophenoxyacetic acid. *Nanotechnology* 23, 495710.

[4] Kerker, M.; Siiman, O.; Bumm, L.A.; Wang, D.S. ***(1980)*** Surface enhanced Raman scattering (SERS) of citrate ion adsorbed on colloidal silver. Applied Optics, 19:24, 4137-4137. https://doi.org/10.1364/AO.19.004137

[5] Munro, C.H.; Smith, W.E.; Garner, M.; Clarkson, J.; White, P.C. ***(1995)*** Characterization of the Surface of a Citrate-Reduced Colloid Optimized for Use as a Substrate for Surface-Enhanced Resonance Raman Scattering. Langmui, 11 (10), 3712-3720. https://doi.org/10.1021/la00010a021
